# Single-Layer Graphene/Germanium Interface Representing a Schottky Junction Studied by Photoelectron Spectroscopy

**DOI:** 10.3390/nano13152166

**Published:** 2023-07-26

**Authors:** Cesar D. Mendoza, F. L. Freire

**Affiliations:** Departamento de Física, Pontifícia Universidade Católica do Rio de Janeiro, Rio de Janeiro 22451-900, RJ, Brazil; lazaro@vdg.fis.puc-rio.br

**Keywords:** graphene, germanium, Schottky junction, ultraviolet photoelectron spectroscopy, X-ray photoelectron spectroscopy

## Abstract

We investigated the interfacial electronic structure of the bidimensional interface of single-layer graphene on a germanium substrate. The procedure followed a well-established approach using ultraviolet (UPS) and X-ray (XPS) photoelectron spectroscopy. The direct synthesis of the single-layer graphene on the surface of (110) undoped Ge substrates was conducted via chemical vapor deposition (CVD). The main graphitic properties of the systems were identified, and it was shown that the Ge substrate affected the electronic structure of the single-layer graphene, indicating the electronic coupling between the graphene and the Ge substrate. Furthermore, the relevant features associated with the Schottky contact’s nature, the energy level’s alignments, and the energy barrier’s heights for electron and hole injection were obtained in this work. The results are useful, given the possible integration of single-layer graphene on a Ge substrate with the complementary metal-oxide-semiconductor (CMOS) technology.

## 1. Introduction

The electronic, optical, and mechanical properties of graphene make it an attractive candidate for integration with modern semiconductor technology [1,2,3,4,5]. Devices constituting 2D/3D materials have given rise to innovative technological possibilities based on the properties of the interface formed and compatibility with the scalable complementary metal oxide semiconductor (CMOS) technology [6,7,8,9,10,11,12]. The fundamental building block of CMOS technology is consolidated on 3D semiconductor materials such as Si, Ge, and GaAs, which have an electronic bandgap at the Fermi level [13]. Fine-tuned selection of the electronic and optical properties allows for the design of transistors, light-emitting diodes (LEDs), and sensors, among other devices [14,15,16,17,18]. High-quality graphene must be located atop these semiconducting surfaces, either by a transfer process or by direct synthesis on the surface, with the latter being more suitable and compatible with the desired scalability in the industry.

In this context, the recent findings on the direct growth of single-layer graphene on a Ge surface and their ongoing progress provide an appropriate roadmap toward a potentially integrable interface [19,20,21,22,23,24,25,26,27,28,29,30,31,32,33,34]. However, one must pay attention to which Ge surface is used, since the graphene’s growth may or may not be accompanied by faceting of the Ge substrate. While it is planar on the surface of Ge (110), it is not on the surface of Ge (100) [21,22,23,24,31,32,33,34]. Although the orientation (100) is exciting for implementation into Si technology, several works about other atomically flat directions have arisen as models for future applications, such as (110). Furthermore, the height of the Schottky barrier formed at the interface is the critical electrical element that would allow the integration of single-layer graphene and Ge into any modern technology. This barrier is easily tunable owing to the density of states of the 2D nature of graphene, making the Fermi level susceptible to the number of carriers injected into or from the semiconductor. In this way, the quality of the single-layer graphene considerably impacts the junction’s properties. Therefore, this type of contact provides an excellent opportunity to study the physical phenomena occurring at the interface formed by 2D and 3D materials. Moreover, it also offers a convenient platform for investigating electronic properties and transport mechanisms that have not yet been elucidated from the point of view of fundamental knowledge [35,36,37,38,39,40,41]. An approach based on ab initio calculations was also used to study the interface involving 2D materials for predicting the structural and electronic properties, as well as the thermal stability of these interfaces [42,43].

Thus far, research has revealed that the structure of the graphene/Ge interface plays an essential role in optoelectronic applications because it avoids the pinning effect of the Fermi energy level. It limits the height of the Schottky potential barrier of the typical 3D metal/Ge junction. It also enables the use of Ge’s high-carrier mobility, near-infrared absorption coefficient, and compatibility with mainstream Si technology. Consequently, increasing the Schottky potential barrier’s height using the graphene/Ge junction is the key to reducing the dark current and improving the sensitivity and signal-to-noise ratio in this class of devices [40,41,42,44,45,46].

The electronic structure of the interface formed by the Ge surface and single-layer graphene synthesized by chemical vapor deposition (CVD) on a Ge (110) substrate was studied. The procedure used ultraviolet and X-ray photoelectron spectroscopy (UPS and XPS, respectively), following a well-established approach to investigating the properties of interfaces formed by 2D/3D materials [47,48,49,50]. In this work, the graphitic properties, alignments of the energy level, dipoles, and the heights of the energy barrier for electron and hole injection at the interface have all been revealed. These findings could be useful for fabricating Ge- and graphene-based devices.

## 2. Materials and Methods

### 2.1. Sample Preparation

A highly orientated pyrolytic graphite (HOPG) with 0.7° ± 0.2° of mosaic spread was supplied by NT-MDT. The HOPG sample was used to identify and compare the graphitic characteristics of single-layer graphene synthesized on a Ge substrate. It was cleaved with adhesive tape in the air and immediately placed in the analytical chamber, while the gold sample was a standard film for surface analysis. Meanwhile, CVD was used to synthesize single-layer graphene on an undoped Ge (110) substrate at semiatmospheric pressures. Both its Raman spectrum and scanning tunneling spectroscopy images were typical of single-layer graphene, as can be seen in the Supplementary Material in Figure S1a,b and Table S1. The undoped Ge (110) had a resistivity of 30 Ω-cm; it was polished on a single side and was supplied by Semiconductor Wafer Inc., Taiwan, China.

The Ge substrates were cleaned and placed in acetone and isopropyl alcohol for 15 min each, followed by rinsing in deionized water for 15 min. Then the substrate was inserted into a quartz tube. The growth environment was a mixture of argon (99.999% purity) and hydrogen (H_2_, 99.999% purity) gases, with the gas flows being 50 sccm and 100 sccm, respectively (sccm, standard cubic centimeters per minute). As a result of the growth atmosphere of CVD, a hydrogen-passivated Ge surface was expected [29]. Once the deposition temperature and pressure had been reached, methane gas (99.999% purity) was introduced into the reactor. The synthesis was carried out at 910 °C at a pressure of 350 Torr, and the time of synthesis was 60 min. Following the growth of the graphene, the system was naturally cooled to room temperature in the same atmosphere used during synthesis [22,28]. Then the samples were transferred to the analytical chamber in the air. The Ge surface was characterized after annealing in a H_2_–Ar atmosphere in the CVD reactor. Then the sample was transferred in the air to the analytical chamber.

### 2.2. Characterization

Photoelectron spectroscopy measurements were performed using a hemispherical analyzer VG Thermo Alpha 110. It was positioned at a 90° angle to the sample’s surface. The X-ray source in the XPS analysis was the Al-Kα line (~1486.6 eV), whereas the photon source in the UPS was a helium lamp (He(I) ~21.22 eV). The measurements were performed under ultrahigh vacuum conditions (a pressure of ~10^−9^ Torr). Using CASA software (Version 2.3.23PR1.0), the Voigt profile and Shirley background line were important parameters for fitting the XPS spectra.

UPS data were collected with biases of 0 V and −5 V applied between the sample and the analyzer. Usually, during the UPS measurements, the first step was to determine the state density near the Fermi level. The latter was to separate the secondary electrons of the sample and the analyzer, which can be seen in Figure S2. That procedure pushes the spectra away from the secondary electrons of the analyzer, which are not accelerated by the potential remaining close to 0 eV kinetic energy. Hence, the secondary electrons of the sample and analyzer were well separated and could be analyzed individually (see Figure S2a,b). The field effect or charging during ultraviolet illumination was negligible. All UPS spectra used the Fermi edge of the gold sample to calibrate the energy scale relative to the Fermi edge of the sample. Likewise, proper calibration of the energy scale was required for the XPS spectra. The procedure relied on measuring the position of the Au 4f_7/2_ core-level peak from metal foils that had previously been sputter-cleaned, removing possible surface contaminants. That procedure was carried out before analysis of the sample and was regularly repeated to correct any errors that accumulate over time. In addition, it was also used as a second method of verifying the lowest energy portion of the valence band spectra for Au in the vicinity of the Fermi level, where the density of the states exhibited a well-defined Fermi energy, which defined the natural “0 eV” on the energy scale [51]. Figure S3 in the Supplementary Material shows the Fermi edge and the full UPS spectra for the Au sample, as well as the XPS spectra associated with the Au 4f level and the survey.

## 3. Results and Discussion

**Figure 1:**
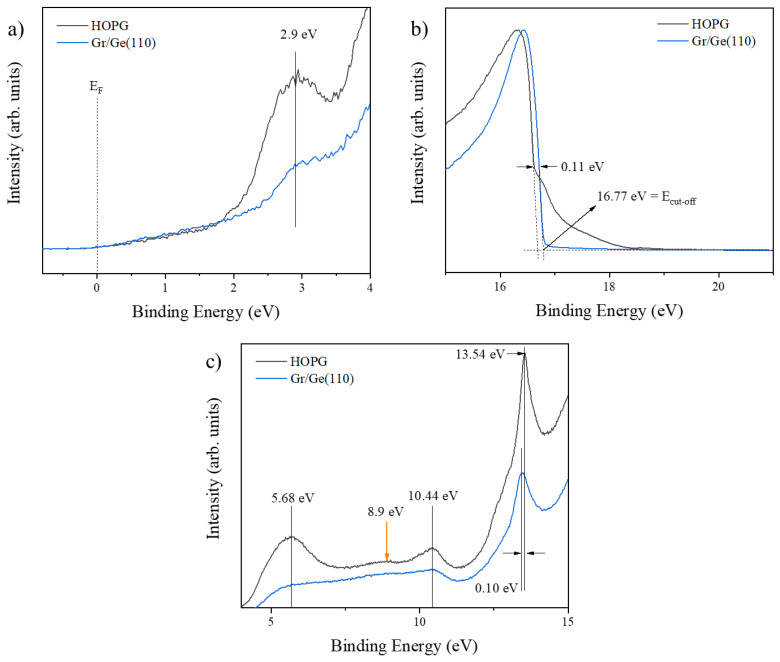
UPS spectra (taken with a bias of −5 V) of the highly oriented pyrolytic graphite (HOPG) and single-layer graphene on a Ge (110) surface (Gr/Ge (110)). The three main regions are the valence band (**a**), the secondary electrons’ cut-off (**b**), and the intermedium range (**c**). The positions of the Fermi level were at 0 eV for each spectrum. The orange arrow at 8.9 eV indicates the σ band from the 2p orbital. All spectra were normalized to make a reliable comparison.

**Figure 2:**
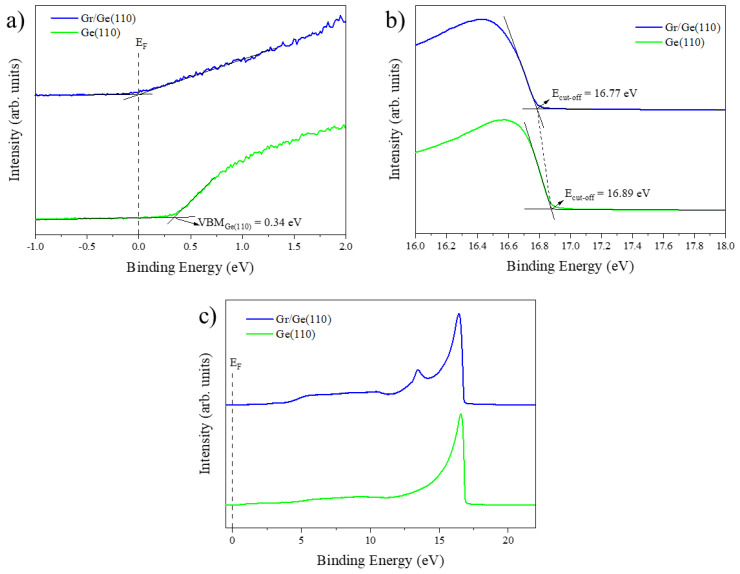
UPS spectra of the interfaces in analysis in this study, taken with a bias of −5 V. The panels show the valence band (**a**), secondary electrons (**b**), and the full UPS spectra (**c**). The VBM and E_cut-off_ from the UPS spectrum of Ge were obtained by extending the baselines and onsets indicated on the spectrum. The reference level was the zero (0 eV) binding energy. The blue spectrum was obtained from the Gr/Ge (110) surface, whereas the green spectra correspond to the Ge (110) substrate.

Figure 1 depicts the UPS spectra (taken with a bias of −5 V) of the highly oriented pyrolytic graphite (HOPG) and the single-layer graphene grown by CVD on the Ge (110) surface (Gr/Ge (110)). It shows three panels that correspond to the valence band (a), the secondary electrons’ cut-off (b), and the intermedium energy range (c). The HOPG surface was used as a reference because it ideally has similar electronic and structural properties to graphene. In the region related to the valence band (Figure 1a), we observed that the Fermi level in the HOPG and the single-layer graphene were at the reference level (0 eV). The method used to determine these energies is shown in Figure 2. The Supplementary Material in Figure S2a,b shows the case of bias of 0 V. The cut-off energies of the HOPG and Gr/Ge (110) in the region of the secondary electrons (Figure 1b) were 16.66 eV and 16.77 eV, respectively. The work function (WF) for each sample can be calculated using Equation (1), where E_cut-off_ is the cut-off energy, E_F_ is the Fermi level, and hυ is the photons’ energy (21.22 eV).

WF = hυ − (E_cut-off_ − E_F_), (1)

The value determined for HOPG was in good agreement with the WF reported in the literature, namely 4.56 eV [47], while the WF of Gr/Ge (110) was 4.45 eV.

Some significant differences existed between the graphene synthesized directly on the Ge substrate and the HOPG. For example, the graphene electrons were at more profound energetic levels at around 0.11 eV (Figure 1b). In addition, graphitic features due to carbon valence electrons were observed and identified using previous results reported in the literature [48]. In Figure 1a, the peak around 2.90 eV is associated with the π electrons of the 2p orbital. In Figure 1c, the peaks observed at 5.68 eV, 10.44 eV, and 13.54 eV are associated with the σ + π band electrons of the 2p orbital, the 2s-2p hybridized state, and the σ band from the 2s orbital, respectively. Simultaneously, the orange arrow at 8.9 eV denotes the σ band from the 2p orbital.

Two key points can be identified from the spectra shown in Figure 1: (1) the graphitic features were more prominent in the HOPG than in the Gr/Ge, and (2) there was a redshift in the energy for some electronic features when comparing the HOPG with the graphene atop the Ge substrate. These observations align with similar studies that partially attributed them to the strong interaction between graphene and the substrates (so-called coupling) [47,52].

In addition, we examined the Ge surface before and after the growth of the single-layer graphene using UPS. Figure 2 depicts the UPS spectra of the samples analyzed in this study. The valence band, secondary electrons, and full UPS spectra of the Ge surfaces and Gr/Ge interfaces are shown in Figure 2a–c, respectively. By extending the baselines and onsets indicated on each spectrum, information about the valence band maximum (VBM) and cut-off energy (E_cut-off_) from the UPS spectrum of Ge was obtained. However, the intrinsic band gap of Ge is 0.67 eV at absolute zero (0 K), and E_F_ is in the middle of the band gap; the VBM of Ge is at 335 meV.

According to Figure 2 and Equation (1), the VBM, E_cut-off_, and WF for Ge (110) were 0.34 eV, 16.89 eV, and 4.33 eV, respectively. In contrast, the WF of Gr/Ge (110) was 4.45 eV. Raman and scanning tunneling spectroscopy (STS) were used to determine whether the single-layer graphene was strained and doped (Figure S1a,c in the Supplementary Material). The shift of the G and 2D bands and the energy difference E_D_ − E_F_ = −65 meV showed that the Gr/Ge system was strained and n-type doped (Figure S1 and Table S1), as previously reported [24,30]. Figure S1b shows an STM image of the single-layer graphene grown on top of a germanium substrate at the atomic scale. The inset in this figure shows the FFT obtained from the STM image, revealing a hexagonal feature typical of single-layer graphene.

**Figure 3:**
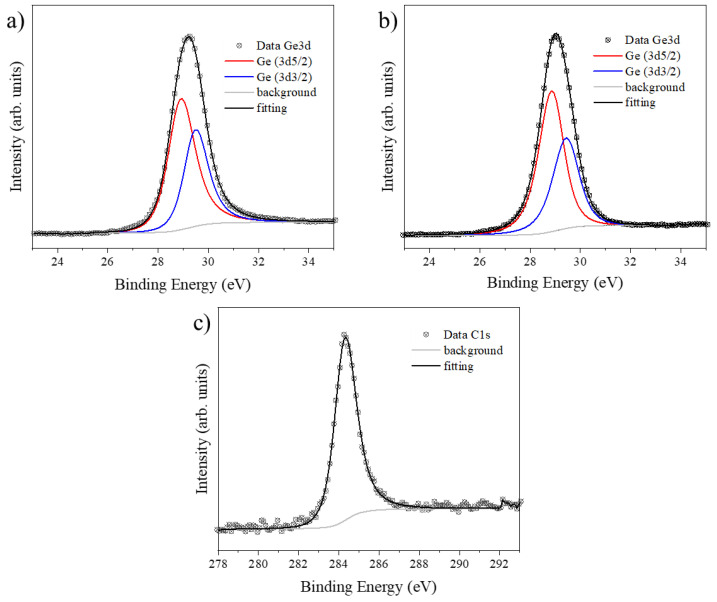
High-resolution spectra obtained by XPS measurements for the Ge3d and C1s peaks. (**a**) The Ge3d peak for the Ge surface (110); (**b**) the Ge3d peak for the Gr/Ge (110) system. (**c**) C1s peak for the Gr/Ge (110) system. The C1s spectra show a single peak of the carbon due to graphene. The contributions of Ge3d_5/2_ and Ge3d_3/2_ are the red and blue lines in each spectrum.

Before and after the growth of graphene, XPS analyses were performed on the Ge surface. Figure 3 depicts the Ge3d and C1s XPS peaks’ core-level spectra. The Ge3d peak had a symmetric line shape and corresponded to an overlap of the doublet of the spin-orbit components (3d_5/2_ and 3d_3/2_ with Δ = 0.58eV and an intensity ratio of 0.67).

**Figure 4:**
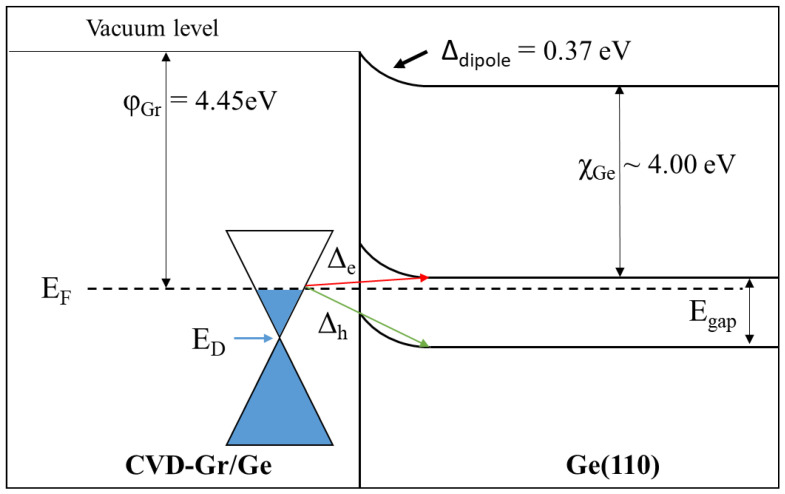
The energy level alignment of the Gr/Ge system is in a single skeleton in thermal equilibrium at zero bias; E_F_ and E_D_ are the Fermi level and Dirac point, while χ and E_gap_ are the germanium’s electronic affinity and band gap, respectively. The hole and electron injection barriers (Δ_h_, green arrow; Δ_e_, red arrow) and the Δ_dipole_ are also indicated. ϕ_Gr_ is the WF of the Gr/Ge system. The representation of the n-type doping of graphene used in this figure is based on the STS characteristics shown in the Supplementary Material (Figure S1b).

The XPS spectra showed that the Ge (110) surface was free of oxide-associated impurities before the growth of graphene, as shown in Figure 3a. They remained free of oxide impurities after deposition of the graphene (Figure 3b). The presence of Ge oxide could be expected due to aging effects caused by the diffusion of oxygen-containing molecules through the graphene’s grain boundaries. However, this occurs only after days of exposure to the environment, but that was not the case [53,54]. It is well known that no germanium–carbon phase is predicted in the Ge-C phase diagram at the synthesis temperature of graphene, since the solubility of carbon in Ge is below parts per billion [19,20,21,22,23,24,25,26,27,28,29,30,31,32,33,34]. These characteristics were confirmed by the XPS spectra of Ge and C, which showed no evidence of C-Ge bonds. As expected, the XPS spectra revealed the presence of carbon due to the graphene (Figure 3c). Two C1s spectra depicted a single component centered at 284.3 ± 0.2 eV (Figure S4 in the Supplementary Material shows the core-level C1s and the survey spectra of the HOPG) associated with the typical graphene synthesized on Ge [31]. The positions of the Ge3d and Ge2p XPS peaks are reported in Table 1. It is worth noting that the more energetic photoelectrons ejected from the inner shell (the Ge2p peak) provided information about the interface, since they were more likely to be affected by impurities. In this case, the position shifts were real. The XPS results showed a shift of 0.3 eV (or 0.1 eV) at the position of the Ge2p peak (or Ge3d). This was used to create the energy level alignment diagrams shown in Figure 4.

The contact Ohmic could be discarded, since the Fermi level of graphene did not overlap with any Ge band, as shown in Figure 4. Conversely, some characteristics of a Schottky contact could be considered, for instance, the barrier height (ϕ_B_), the contact potential (V_0_), and the type of Schottky contact behavior. Because WF(Gr) > WF(Ge), the Schottky−Mott rule proposes that the barrier height is the difference between WF(Gr) and χ_Ge_, while the contact potential is that between WF(Gr) and WF(Ge) [35]. Based on the values above, the Gr/Ge (110) system has a ϕ_B_ of ~0.46 eV and a V_0_ of ~0.12 eV. On the other hand, UPS combined with XPS allowed us to determine accurate band alignments at the Gr/Ge interface, as shown in Figure 4. For that, it was necessary to know where the maximum valence band of the substrate was. However, its position could not be directly detected from the UPS due to the additional density of the states from single-layer graphene. However, it was possible to find out the VBM indirectly from the core level of Ge2p. Knowing the positions before and after the coverage of the Ge substrate, shown in Table 1, and the VBM of Ge (110) without graphene (0.34 eV), we could deduce where the VBM of Gr/Ge (110) was. This was because the distance between Ge2p and VBM was constant (1218.2 eV).

Figure 4 summarizes the interfacial energy level of the Gr/Ge (110) system in a single skeleton. We calculated the electronic affinity (χ) of the Ge substrates by combining the WF and the VBM of pure Ge substrates with the bandgap (E_gap_ = 0.68 eV) of Ge at room temperature. Due to the band alignments, the vacuum level shifted upward due to the formation of a negative surface dipole (Δ_dipole_ = −0.37 eV). The hole and electron injection barriers (Δ_h_, green arrow; Δ_e_, red arrow in Figure 4) could be calculated by considering E_F_ (Gr/Ge), WF (Gr/Ge), the Δ_dipole_, χ_Ge_, and E_gap_ (Ge). The Δ_e_ for single-layer graphene on top of Ge (110) is around 0.08 eV, while the Δ_h_ is around 0.60 eV. The results showed that Δ_h_ was higher than Δ_e_, indicating that the single-layer graphene synthesized directly on Ge substrates is suitable for injecting electrons into the semiconducting material. The comparison between our band alignments from the UPS and XPS results and those resulting from the Schottky–Mott rule lets us see that the rule is not perfect.

A previous investigation using scanning probe microscopy techniques reported that a heterojunction formed by CVD graphene and n-doped Ge (100) was a Schottky junction with the height of the energy barrier (ϕ_B_) being 0.45 eV. They also indicated n-type doping of the graphene layer with a Fermi level of 0.15 eV above the Dirac point [41].

In order to compare our results with previously published results, we added Table 2. Thus, our results (shown above) are partly in line with those of others. However, it is important to mention that this table lists several studies reporting Schottky barrier values obtained from I–V curves of graphene transferred to Ge surfaces. Our work derived this value from the photoelectron spectroscopy data of graphene samples grown directly on Ge by CVD. It is important to note that the procedures of graphene transfer frequently result in residual polymer contamination on top of the graphene surface. It is not easy to imagine that the popular transfer procedures used in the abovementioned articles could be implemented in a modern production line making devices.

## 4. Conclusions

In summary, we used photoelectron spectroscopy measurements to investigate the interfacial electronic structure of graphene/Ge systems. They demonstrated that the Ge substrates influence the electronic structure of single-layer graphene. The interaction between the single-layer graphene and the Ge substrates was attributed to a slight difference in the WFs between the Gr/Ge and the HOPG samples, in addition to the absence of Ge features in the UPS spectra. The redshift in some graphitic characteristics in the UPS spectra also supported the interaction (or coupling) between graphene and Ge. Finally, we calculated the total interfacial energy level alignments of graphene/Ge (110) by considering the E_F_ (Gr/Ge), WF (Gr/Ge), and the amount of Δ_dipole_, χ_Ge_, and E_gap_ (Ge). Our results showed that the single-layer graphene synthesized directly on Ge substrates is suitable for injecting electrons into a semiconducting material. This information could be helpful in optoelectronic applications where the graphene would work as a transparent electrode, facilitating the transport of n-type charge carriers.

## Figures and Tables

**Table 1:** Positions of the main XPS peaks for each sample, with an uncertainty of ±0.2 eV.

Sample	Positions of the Peak (eV)			
	Ge3d_5/2_	Ge2p_3/2_	C1s	O1s
Ge (110)	28.9	1218.5	---	---
Gr/Ge (110)	28.8	1218.8	284.3	---

**Table 2:** Comparison of our results with previously published results.

Substrate	Interface	SBH (eV)	Building of the Interface ^@^	Determination	Reference
n-Ge	Gr/Ge	0.11 and 0.70	Transfer	I vs. V	[44]
n-Ge	Gr/Ge	0.40	Transfer	I vs. V	[45]
n-Ge	Gr/Ge	0.53	Transfer	I vs. V	[46]
p-Ge	Gr/Ge	0.45	Direct	C-AFM *	[41]
Ge	Gr/Ge	0.30	Transfer	---	[40]
Ge	Gr/Ge	0.46 **	Direct	XPS and UPS	This work

* C-AFM is the conducting atomic force microscopy measurement, using the AFM tip (conducting) on a Gr/Ge interface to obtain the I vs. V curve. ** In our work, the SBH was obtained using the Schottky–Mott rule. ^@^ The Gr/Ge interface can be built by transfer and direct growth.

## Data Availability

The details regarding the data supporting reported results can be obtained from C. D. Mendoza upon direct request.

## Supplementary Materials

The following supporting information can be downloaded at: https://www.mdpi.com/article/10.3390/nano13152166/s1, Figure S1: (a) Full Raman spectra of the Gr/Ge systems: the left-hand diagram corresponds to Gr/Ge(100), while the right-hand diagram corresponds to Gr/Ge(110). The main bands are the D- (~1370 cm^−1^), G- (~1590 cm^−1^), and 2D-bands (~2720 cm^−1^). The spectra also show the O2 (~1554 cm^−1^) and N2 (~2329 cm^−1^) peaks. (b) The honeycomb structure of graphene/Ge obtained by STM, showing that our interface was built from a graphene monolayer. The insert is the FFT obtained from the STM image in (b). (c) STS measurements at the Gr/Ge (110) interface. E_F_ is the Fermi level (dashed line), and ED is the Dirac point (orange arrow), where ED − EF = −65 meV; Table S1: Positions of the bands of the Raman spectra of single-layer graphene under two conditions; Figure S2: Full UPS spectra, taken with a bias of 0 V (a) and −5 V (b), of the highly oriented pyrolytic graphite (HOPG, black line) and the single-layer graphene on the Ge (110) surface (Gr/Ge (110), blue line). The positions of the Fermi level of the spectrometer were adjusted using a gold (Au) sample, E_Reference_ (0 eV). EF_HOPG and E_F__Gr/Ge (110) are the Fermi levels corresponding to 0 eV. The orange arrow at 8.9 eV indicates the σ band from the 2p orbital, while the black arrows indicate the region of the secondary electrons. All spectra were normalized to make a reliable comparison among them. UPS spectra of the region of the valence band were taken with a bias of 0 V; the Gr/Ge (110) is in (c). Moreover, (c) shows the valence band maximum (VBM) and Fermi levels obtained from the UPS spectra of Ge and Gr/Ge by extending the baselines and onsets, as indicated on each spectrum. The Au reference level is the zero (0 eV) binding energy. The blue spectrum was obtained from the Gr/Ge (110) system, while the green spectrum corresponds to Ge (110) surfaces; Figure S3: (a) The Fermi edge for the Au sample. Red lines are associated with the extrapolation method to obtain the Fermi level. (b) Full UPS spectrum of the Au sample. The position of the Fermi level (indicated with a dashed black line) concerning the analyzer was adjusted and indicated as the zero binding energy (0 eV). (c) XPS core-level spectrum for the Au4f at 84.0 eV; (d) survey spectrum. In (c), the Au4f peak shows spin-orbital components (4f_7/2_ and 4f_5/2_) corresponding with the state Au0. Au4f_7/2_ and Au4f_5/2_ are the solid and dashed orange lines for Au0, respectively. (e) The energy distribution around EF (0 eV) in a UPS spectrum from an Au sample at 300 K (solid black line). The resolution ΔE was obtained by convoluting a Fermi function (T = 300 K, green dashed line) with a Gaussian function with a width of ΔE = 53 meV (FWHM); Figure S4: XPS measurements for the HOPG sample: (a) XPS core-level spectrum for the C1s; (b) survey spectrum. In (a), the position of the carbon peak was 284.3 ± 0.2 eV; Figure S5: XPS measurements for the Ge substrate and Gr/Ge system are shown: (a,c) are the survey spectra, while (b,d) are the Ge2p spectra. References [24,55] are cited in the Supplementary Materials.
