# Supplementary material for: Single-Layer Graphene/Germanium Interface Representing a Schottky Junction Studied by Photoelectron Spectroscopy
Source: Nanomaterials (Basel). 2023 Jul 26;13(15):2166. doi: 10.3390/nano13152166 (PMC10420948; doi:10.3390/nano13152166)
Supplement: Supplementary file 1 [file nanomaterials-13-02166-s001.zip › nanomaterials-2515904-supplementary.pdf]

Supplementary Material

Single-Layer Graphene/Germanium Interface Representing a Schottky Junction Studied by Photoelectron Spectroscopy

Cesar D. Mendoza * and F. L. Freire, Jr.

Departamento de Física, Pontifícia Universidade Católica do Rio de Janeiro, Rio de Janeiro 22451-900, RJ, Brazil; lazaro@vdg.fis.puc-rio.br
* Correspondence: cesar.diaz@vdg.fis.puc-rio.br

Characterization:

The Raman measurements were obtained using a micro-Raman spectrometer (NT-MDT, NTEGRA SPECTRA) equipped with a charge-coupled device detector and a solid-state laser. The wavelength of the laser excitation was 473 nm (2.62 eV). We used a 100X magnification objective and an incident laser power of less than 0.2 mW to create a laser spot with an area of approximately 1.0 μm^2. Scanning tunneling spectroscopy (STM) was performed at room temperature in a UHV Omicron™ microscope at 10-8 Pa pressure using electrochemically etched tungsten (W) tips.

The Raman spectrum in Figure S1a) shows the main graphene bands, the D- (~1373 cm^{-1}), G- (~1590 cm^{-1}), and 2D- (~2741 cm^{-1}) bands. They show peaks due to O_2 (~1554 cm^{-1}) and N_2 (~2329 cm^{-1}) presence from the atmospheric environment, and these positions are used as an internal standard for energy calibration. The I_{2G}/I_D ratio is 2.5, and the FWHM of the 2G band is around 32 cm^{-1}, typical of single-layer graphene. Also, the honeycomb structure of graphene/Ge obtained by STM shows that our interface is built from a graphene monolayer. The insert is FFT obtained from the STM image in b). The position of the G and 2D bands indicates that the system Gr/Ge has strain status. On the other hand, the representative STS measurement in Figure S1c) shows the characteristic of the interface Gr/Ge (110), where there is a behavior n-type poping, with positions $E_D - E_F$ = -65 meV. E_F and E_D are the Fermi level (dashed line) and Dirac point (orange arrow).

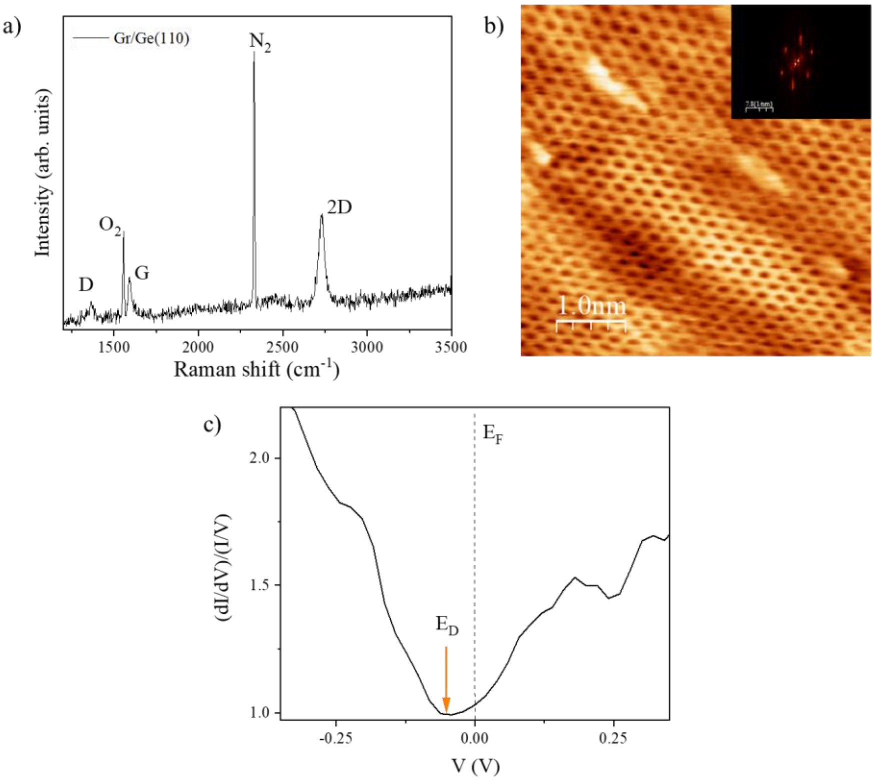

Figure S1. (**a**) Full Raman spectra of Gr/Ge systems: the left hand corresponds to Gr/Ge(100) while the right hand corresponds to Gr/Ge(110). The main bands are the D- (~1370 cm^{-1}), G- (~1590 cm^{-1}), and 2D- bands (~2720 cm^{-1}). The spectra also show the O_2 (~1554 cm^{-1}) and N_2 (~2329 cm^{-1}) peaks. (**b**) the honeycomb structure of graphene/Ge obtained by STM shows that our interface is building from a graphene monolayer. The insert is FFT obtained from the STM image in (**b**). (**c**) STS measurements at the Gr/Ge (110) interface, E_F is Fermi level (dashed line), and E_D is Dirac point (orange arrow), where $E_D - E_F$ = -65 meV.

The graphene band positions related to the Raman spectrum are sensitive to doping and strain levels. Table S1 depicts the comparison among the positions of the Raman bands of single-layer graphene in two conditions using the laser line of 473nm (2.62eV); (1) free-standing [55] and (2) graphene coupled to Ge substrate [24].

Table S1. Positions of the bands of the Raman spectra of single-layer graphene under two conditions.

Band	*Graphene free-standing*	*Graphene coupling*
G (cm^{-1})	1581	1592
2D (cm^{-1})	2693	2724
I(2D)/I(G)	3.1	2.5

Figure S2a,b shows full UPS spectra taken with the bias of 0 V (a) and -5 V (b) of the highly oriented pyrolytic graphite (HOPG, black line), single-layer graphene on the single-layer graphene atop the Ge(110) surface (Gr/Ge(110), blue line). Figure S2a can be seen the overlap between the graphitic feature at 13.64 eV and secondary electrons, while Figure 2b shows the separation of these two regions. Figure S2c shows the region of the valence band of Ge substrates (green lines) and systems Gr/Ge (blue line) obtained with a bias of 0 V. The Fermi levels are the same and correspond to 0 V, while VBM for Ge(110) was 0.34 eV.

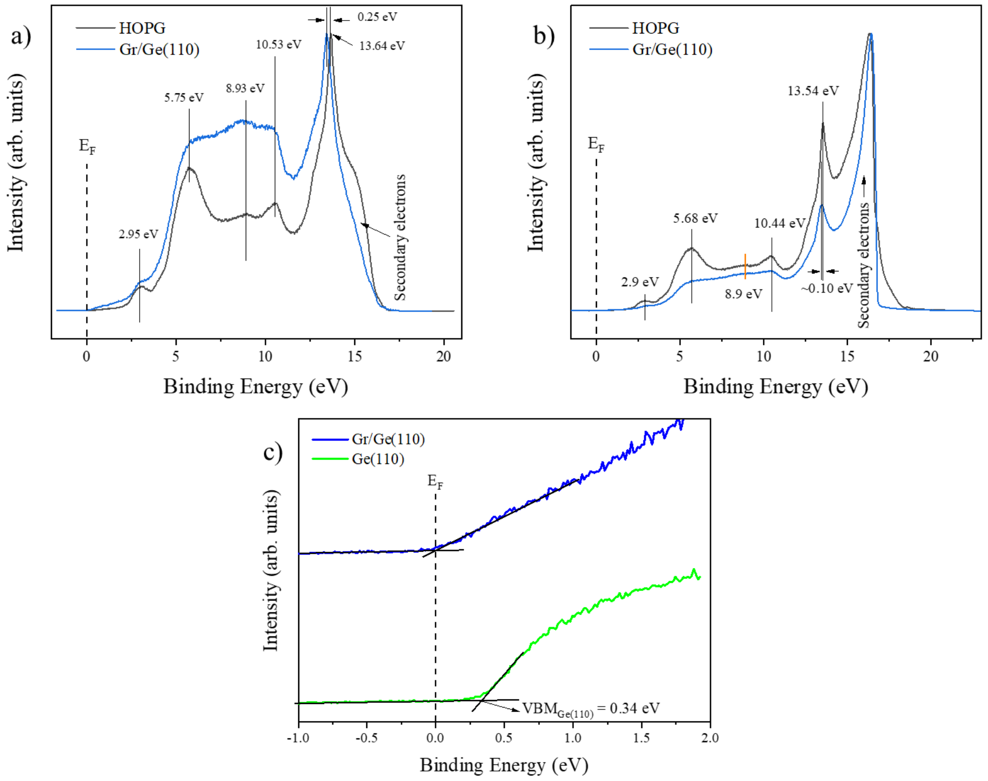

Figure S2. UPS full spectra were taken with the bias of 0 V (**a**) and -5 V (**b**) of the highly oriented pyrolytic graphite (HOPG, black line), single-layer graphene on the Ge(110) surface (Gr/Ge(110), blue line). The Fermi level positions of the spectrometer were adjusted using the gold (Au) sample, $E_{Reference}$ (0 eV). EF_HOPG and EF_Gr/Ge(110) are Fermi levels corresponding to 0 eV. The orange arrow at 8.9 eV indicates the σ band from the 2p orbital, while the black arrows indicate the region of secondary electrons.

All spectra were normalized to make a reliable comparison among them. UPS spectra of the region valence band were taken with a bias of 0 V; the Gr/Ge(110) is in (**c**). In (**c**) is shown the valence band maximum (VBM) and Fermi levels obtained from UPS spectra of Ge and Gr/Ge by extending the baselines and onsets as indicated on each spectrum. The Au reference level is the zero (0 eV) binding energy. The blue spectrum was obtained from the Gr/Ge(110) system, while the green spectrum corresponds to Ge(110) surfaces.

A gold film was used as a reference for calibrating the Fermi level concerning the analyzer. Figure S3a depicts the Fermi edge of the Au film after adjusting the energy scale. Figure S3b corresponds to the full UPS spectrum of the Au film, where features are indicated as Fermi level (E_F), *5d* band, and secondary electrons cut-off ($E_{cut-off}$). Figure S3c,d correspond to the core-level (Au*4f*) and survey spectra obtained by XPS measurements on the Au film. The energy in the XPS spectra was calibrated using the Fermi level obtained by UPS on the Au sample and the position of the Au $4f_{7/2}$ core-level peak at 84.0 eV.

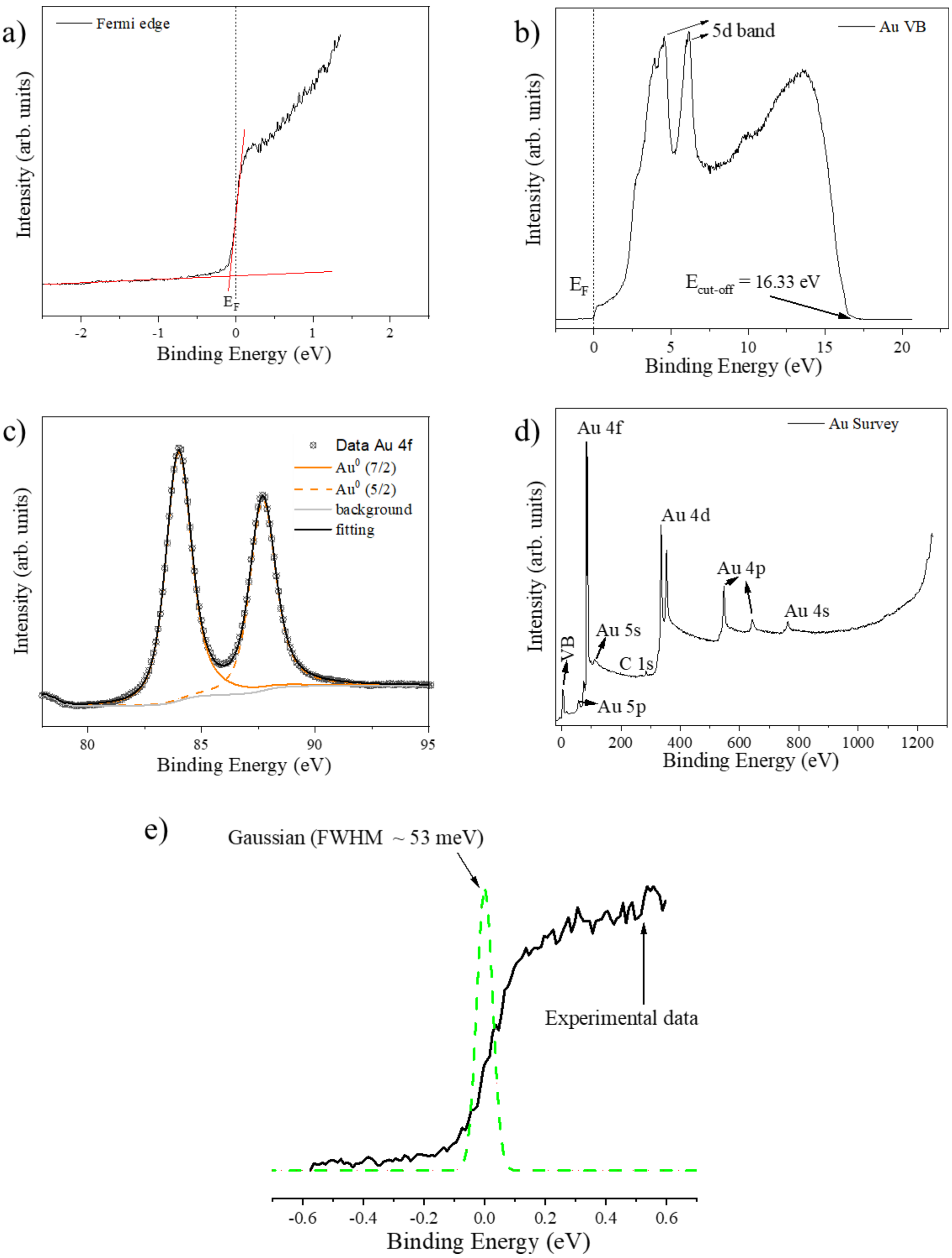

Figure S3. (**a**) the Fermi edge for the Au sample. Red lines are associated with the extrapolationmethod to obtain the Fermi level; (**b**) UPS full spectrum of the Au sample. The Fermi level position indicated with a dashed black line concerning the analyzer was adjusted and indicatedas the zero binding energy (0 eV); (**c**) XPS core-level spectrum for the Au4*f* at 84.0 eV; (**d**) survey spectrum. In (**c**), the Au4*f* peak shows spin-orbital components (4*f*7/2 and 4*f*5/2) corresponding with the states Au^0. Au4*f*7/2 and Au4*f*5/2 are the solid and dashed orange lines for Au^0, respectively. (**e**) Energy distribution around E_F (0 eV) in a UPS spectrum from an Au sample at 300 K (solid black line). The resolution ΔE is obtained by convoluting a Fermi function (T = 300 K, green dashed line) with a Gaussian function of width ΔE = 53 meV (FWHM).

Figure S4 depicts the XPS core-level spectrum for the C1s and survey spectrum measured from a HOPG sample. In the main text, we use the Ge*3d* peak to illustrate the XPS spectra of Ge rather than the Ge*2p* peak. Figure S5 shows the survey spectra and the Ge*2p* peaks to low kinetic energy electrons obtained by XPS for each sample.

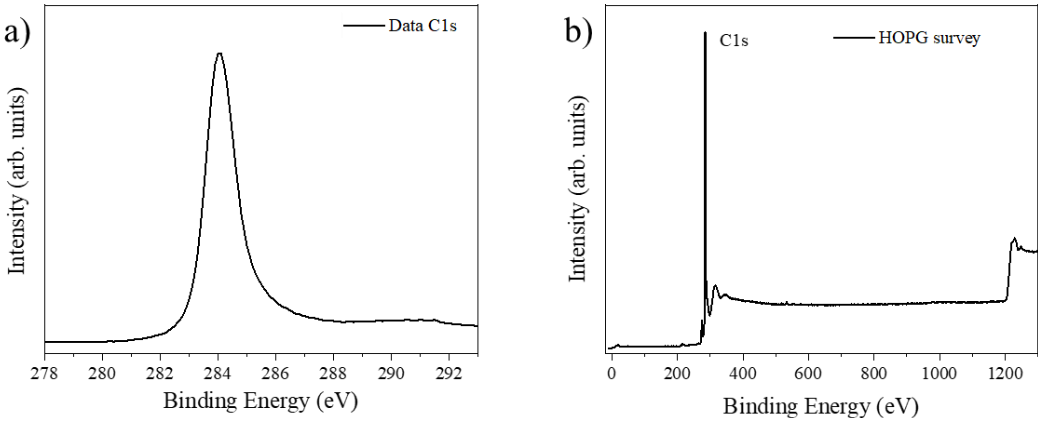

Figure S4. XPS measurements for the HOPG sample: (**a**) XPS core-level spectrum for the C*1s*; (**b**) survey spectrum. In (**a**), the position of the carbon peak was 284.3 ± 0.2 eV.

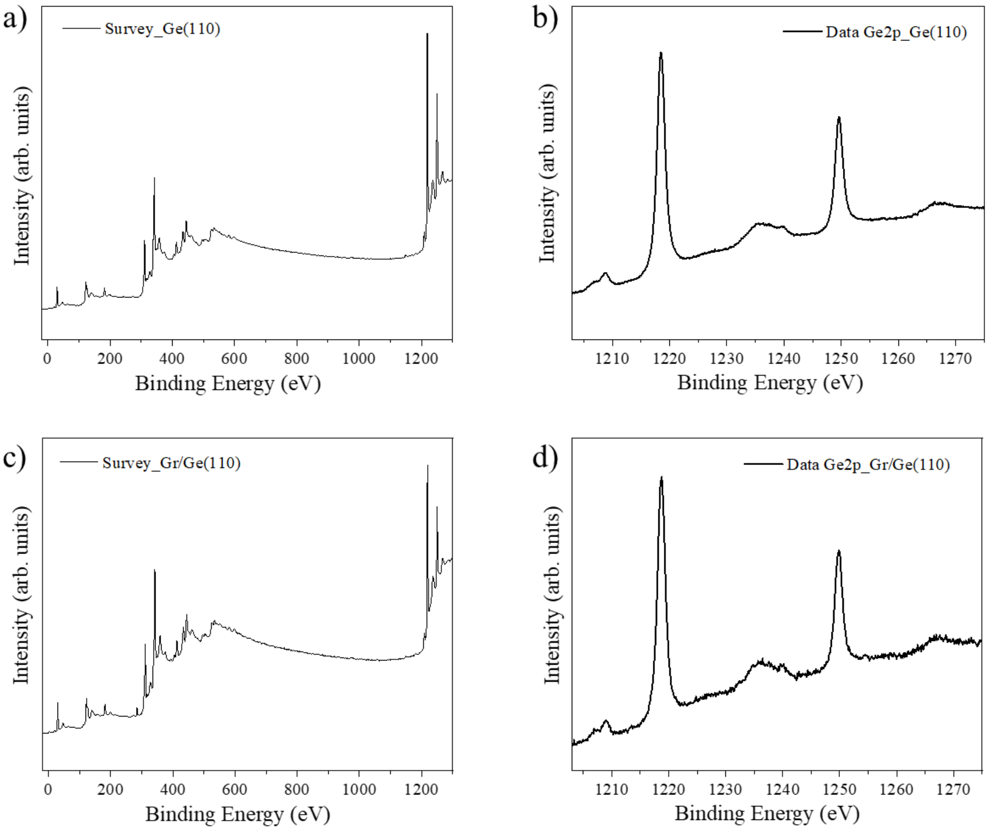

Figure S5. XPS measurements for the Ge substrate and Gr/Ge system are shown: (**a**,**c**) are the survey spectra, while (**b**,**d**) are Ge*2p* spectra.

References

24. C.D. Mendoza, P.G. Caldas, F.L. Freire, M.E.H. Maia Da Costa, Growth of single-layer graphene on Ge (1 0 0) by chemical vapor deposition, Applied Surface Science. 447 (2018) 816–821. https://doi.org/10.1016/j.apsusc.2018.04.019.
55. S. Berciaud, X. Li, H. Htoon, L.E. Brus, S.K. Doorn, T.F. Heinz, Intrinsic Line Shape of the Raman 2D-Mode in Freestanding Graphene Monolayers, Nano Lett. 13 (2013) 3517–3523. https://doi.org/10.1021/nl400917e.
